# Whole-Exome Sequencing Reveals Rare Genetic Variants in Saudi COVID-19 Patients with Extreme Phenotypes

**DOI:** 10.3390/v17091198

**Published:** 2025-08-30

**Authors:** Rashid Mir, Mohammad Fahad Ullah, Imadeldin Elfaki, Mohammad A. Alanazi, Naseh A. Algehainy, Faisal H. Altemani, Mamdoh S. Moawadh, Faris J. Tayeb, Badr A. Alsayed, Mohammad Muzaffar Mir, Jaber Alfaifi, Syed Khalid Mustafa, Jameel Barnawi, Salma Saleh Alrdahe

**Affiliations:** 1 Prince Fahad Bin Sultan Chair for Biomedical Research, University of Tabuk, Tabuk 71491, Saudi Arabia; nalgehainy@ut.edu.sa (N.A.A.); jbarnawi@ut.edu.sa (J.B.); 2 Department of Medical Lab Technology, Faculty of Applied Medical Sciences, University of Tabuk, Tabuk 71491, Saudi Arabia; m.alenezi@ut.edu.sa (M.A.A.); faltemani@ut.edu.sa (F.H.A.); mmoawadh@ut.edu.sa (M.S.M.); f.tayeb@ut.edu.sa (F.J.T.); 3 Department of Biochemistry, Faculty of Science, University of Tabuk, Tabuk 71491, Saudi Arabia; elfakiimadeldin@gmail.com; 4 Department of Internal Medicine, Faculty of Medicine, University of Tabuk, Tabuk 71491, Saudi Arabia; balsayed@ut.edu.sa; 5 Department of Clinical Biochemistry, College of Medicine, University of Bisha, Bisha 61922, Saudi Arabia; mmmir@ub.edu.sa; 6 Department of Child Health, College of Medicine, University of Bisha, Bisha 61922, Saudi Arabia; jalfaifi@ub.edu.sa; 7 Department of Chemistry, Faculty of Science, University of Tabuk, Tabuk 71491, Saudi Arabia; ssyed@ut.edu.sa; 8 Department of Biology, Faculty of Science, University of Tabuk, Tabuk 71491, Saudi Arabia; salrdahe@ut.edu.sa

**Keywords:** SARS-CoV-2, whole-exome sequencing, genetic variants, comorbidity

## Abstract

The global impact of COVID-19 was staggering, with millions of cases and related mortality reported worldwide. Genetic variations play a significant role in determining an individual’s susceptibility to SARS-CoV-2 infection and progress to severe disease. This pilot study provides an experimental approach using WES to identify certain rare and novel genetic variants that might affect an individual’s susceptibility to the risk of SARS-CoV-2 infection, offering an initial exploration of these genetic variants. In the study cohort with 16 patients, the mortality rate was higher in male patients due to severe disease. There was a substantial burden of comorbidity, including hypertension, ischemic heart disease, and T2DM, conditions which independently increase the risk of adverse outcomes in COVID-19 patients. A total of 4478 variants were identified, distributed across 322 genes within the cohort. The majority of these variants were missense substitutions along with frameshift variants, inframe insertions/deletions (indels), and nonsense variants. The variants were further categorized by types to include single-nucleotide polymorphisms (SNPs), deletions (DEL), and insertions (INS). The gene with the highest number of variants was *HLA-DRB1*, followed by *HLA-B*, *ABO*, *HPS4*, and *SP110* displaying both common polymorphisms and rare variants. Moreover, the *HLA-B* gene exhibited the highest number of rare candidate variants, followed by *AK2*, *IRF7*, *KMT2D*, *TAP1*, and *HLA-DRB1*. Several genes harbored multiple novel variants, including *TAP1*, *AK2*, *G6PC3*, *HLA-B*, *IL12RB2*, and *ITGB2*. The frequencies of the identified variants were found to be either zero or extremely low (below 1% threshold) in the Middle Eastern or in the overall combined population, suggesting that these are indeed rare and do not represent common indigenous polymorphisms. Functional enrichment analysis of the constructed protein–protein interaction network in our preliminary findings revealed that the identified genes are primarily enriched in pathways associated with immune deficiency and DNA repair. This initial exploration of genetic variants in COVID-19 susceptibility provides a foundation for future large-scale studies.

## 1. Introduction

The emergence of COVID-19 in 2020 marked a significant turning point in global health, with millions of lives lost in the pandemic across the world [1]. As of January 2025, the global impact of COVID-19 was staggering, with over 777 million cases reported worldwide leading to more than 7.1 million deaths [2]. The outbreak was initially linked to the Huanan wholesale seafood market in Wuhan, suggesting a potential zoonotic origin [3]. The causative agent of this novel pneumonia was identified as a new type of coronavirus, named SARS-CoV-2. This virus belongs to the genus Betacoronavirus, a group that also includes other notable viruses such as OC43, HKU1, SARS-CoV, and MERS-CoV [4]. The basic reproduction number (R0) of SARS-CoV-2 is estimated to be between 2 and 2.5, indicating its high transmissibility [5]. The disease tends to be more severe in the elderly population, and individuals with comorbidities such as diabetes mellitus, hypertension, and coronary artery disease [6]. These groups often have weakened immune systems or underlying health conditions that make them more vulnerable to the virus. SARS-CoV-2 uses ACE2 receptor as its primary entry point into cells [7]. The ACE2 receptor is found in various tissues throughout the body, including the lungs, heart, kidneys, and gastrointestinal tract. This widespread distribution of ACE2 receptors explains the multi-organ involvement seen in COVID-19 due to the systemic inflammatory response triggered by the virus. The virus binds to the ACE2 receptor on the surface of host cells, allowing it to enter and initiate the replication process. In some individuals, the immune response to SARS-CoV-2 can become dysregulated, leading to a cytokine storm, characterized by the overproduction of inflammatory cytokines. This excessive inflammation can cause damage to the lungs and other organs, leading to acute respiratory distress syndrome and respiratory failure [8]. Genetic variations play a significant role in determining an individual’s susceptibility to SARS-CoV-2 infection [9]. These variations influence how the virus interacts with host cells, affecting the efficiency of viral entry, replication, and the subsequent immune response. Understanding these genetic factors that engage the complex interplay between host genetics and viral pathogenesis are crucial for identifying individuals at higher risk of infection, thereby enabling the development and implementation of targeted preventive strategies. Molecular genetic markers, particularly single-nucleotide variants, are regions of interest within the human genome that can influence gene expression or protein functionality, contributing to differential disease susceptibility. For instance, ACE2, a critical receptor for SARS-CoV-2, and TMPRSS2 which is related to spike protein exhibits numerous genetic variations that influence viral binding affinity and host cell entry [10]. Similarly, variants of immune-related genes such as IFITM3 and IL-6, can affect the host’s ability to mount an effective antiviral response, influencing the severity and outcome of infection [11]. Whole-Exome Sequencing (WES) is a pivotal genomic technique specifically targeting the exome, which comprises exons, the functional segments of genes that encode proteins [12]. Compared to genome-wide association studies, WES exhibits higher sensitivity in identifying low-frequency and rare mutations. This current exploratory study aims to identify genetic predispositions or exacerbating factors that may influence an individual’s susceptibility to, or the severity of, SARS-CoV-2 infection. Utilizing an experimental approach with Whole-Exome Sequencing (WES), we sought to preliminarily identify certain rare and novel genetic variants potentially associated with these outcomes. It is crucial to clarify that the primary aim of this study was not to identify genetic variants causing diseases following strict Mendelian inheritance patterns. Instead, our prioritization focused specifically on identifying variants hypothesized to influence an individual’s response to SARS-CoV-2, impacting either their susceptibility to infection or the progression to severe disease. Within our COVID-19 study cohort, a number of rare candidate variants were subsequently classified as either pathogenic or likely pathogenic, based on the established guidelines of the American College of Medical Genetics and Genomics (ACMG).

## 2. Methodology

### 2.1. Study Subjects and Selection Criteria

This case series study investigated a specific cohort of patients afflicted with severe COVID-19, aiming to unravel the underlying genetic predispositions contributing to extreme disease phenotypes. From a larger pool of 115 COVID-19 patients admitted to two prominent tertiary care hospitals in Saudi Arabia—King Fahad Hospital in Tabuk City and King Abdullah Hospital in Bisha City—a focused subgroup of 16 unrelated individuals was carefully chosen. These patients were recruited between February 2021 and June 2021, a period marked by significant SARS-CoV-2 prevalence.

The selection of these 16 patients was based on their severe COVID-19 symptoms. Notably, 75% of them needed to be admitted to Intensive Care Units (ICUs). The inclusion criteria were defined to ensure consistency within the selected cohort. Patients had to be middle-aged to old-aged, specifically above 35 years, reflecting a demographic often associated with increased COVID-19 severity. A definitive diagnosis of SARS-CoV-2 infection was paramount, confirmed by a positive result on real-time reverse transcription-quantitative polymerase chain reaction (RT-qPCR) using nasal and oropharyngeal swab specimens SARS-CoV-2 RT-qPCR detection kit (Bio Rad, Hercules, CA, USA). Crucially, all included patients presented with acute respiratory distress syndrome (ARDS), a life-threatening complication of COVID-19, regardless of their outcome (survivors or deceased). To mitigate potential confounding factors, individuals who had received any SARS-CoV-2 vaccination prior to admission were excluded, ensuring that the observed phenotypes were purely a result of natural infection and host response. Conversely, patients under 35 years of age were systematically excluded from the study.

A comprehensive, structured questionnaire was administered to all enrolled subjects to gather a rich array of epidemiological and demographic data. This included detailed information on their medical history, specifically focusing on pre-existing conditions known to influence COVID-19 severity, such as coronary artery disease (CAD), type 2 diabetes mellitus (T2DM), and chronic kidney disease (CKD). Furthermore, the questionnaire explored lifestyle factors, including addiction habits, with particular attention to smoking history. A thorough family history of other significant illnesses was also documented to provide a holistic view of potential genetic predispositions.

For Whole-Exome Sequencing (WES), a critical component of this genetic investigation, a peripheral blood sample of approximately 2 mL was collected from each patient. These samples were drawn into EDTA tubes to preserve DNA integrity and immediately stored at −20 °C to prevent degradation until subsequent analysis.

The ethical conduct of this study was of utmost importance. All procedures adhered strictly to regional regulations and were broadly compliant with the foundational principles of the Helsinki Declaration. Ethical approvals were meticulously secured from the institutional ethics committees at the University of Bisha (Ref. no. UBCOM/H-06-BH-087(05/25)) and King Khalid University, Abha (Ref. no. H-06-B-091). Furthermore, explicit written informed consent was obtained from every participant before their involvement in the study, underscoring the commitment to patient autonomy and ethical research practices.

### 2.2. Genomic DNA and Library Preparation

High-quality human genomic DNA was used as the starting material for this study. Exome capture libraries were generated using the Twist Exome Panel 2.0 kit (Twist Bioscience, South San Francisco, CA, USA), following the manufacturer’s recommended protocol. This panel is specifically designed to capture a total of 36.8 Mb of human protein-coding genes. This process involved the following steps: enzymatic fragmentation of genomic DNA to produce fragments of optimal size for sequencing; ligation of specific adapter sequences to the DNA fragments to facilitate sequencing; hybridization of the adapter-ligated fragments to a pool of biotinylated oligonucleotide probes targeting human exonic regions; capture of the hybridized fragments using streptavidin-coated magnetic beads; and polymerase chain reaction (PCR) amplification to enrich the captured exonic DNA fragments, thereby creating the sequencing library. The resulting libraries were quantified using the Qubit dsDNA HS Assay Kit (Thermo Fisher Scientific, Waltham, MA, USA), and library fragment size distribution was assessed using an Agilent 2100 Bioanalyzer (Agilent Technologies, Santa Clara, CA, USA).

### 2.3. Next-Generation Sequencing

Sequencing was performed on the Illumina NovaSeq 6000 platform (Illumina, San Diego, CA, USA) according to the manufacturer’s recommendations. The prepared exome capture libraries were loaded onto the flow cell at the recommended concentration, and paired-end sequencing was performed. The specific sequencing parameters, including read length (2 × 150 bp) and index read configuration, were set according to the Twist 2.0 Exome kit protocol to ensure optimal data generation. Raw sequencing data was generated in FASTQ format.

### 2.4. Quality Control and Preprocessing of Sequencing Reads

The quality of the raw sequencing reads was assessed using FastQC v0.11.9 [13]. This software provides a comprehensive quality report, including metrics such as per-base sequence quality scores, per-sequence quality scores, GC content distribution, adapter content, and sequence duplication levels. Subsequently, adapter sequences and low-quality bases were removed from the raw reads using TrimGalore v0.6.6 [14]. TrimGalore is a wrapper tool that employs Cutadapt [15] to perform adapter trimming and quality filtering.

### 2.5. Read Mapping

The high-quality, trimmed reads were mapped to the hg38 human reference genome assembly using the Burrows–Wheeler Aligner (BWA-MEM) version 0.7.17 [16]. BWA-MEM is an efficient algorithm for aligning short reads to large reference genomes. The alignment process generated Sequence Alignment/Map (SAM) files, which were then converted to the binary BAM format using SAMtools version 1.9 [17] for efficient storage and processing.

### 2.6. Variant Discovery and Annotation

Variant discovery was performed using the Genome Analysis Toolkit (GATK) v4.3 [18], following the GATK Best Practices workflow. Specifically, the HaplotypeCaller tool was employed to identify small genetic variants, including single-nucleotide variants (SNVs) and short insertions/deletions (InDels). HaplotypeCaller assembles reads into local de novo haplotypes, which improves the accuracy of variant calling, especially in complex genomic regions. Comprehensive variant annotation was performed to characterize the identified variants. The RefSeq database [19] was utilized to identify and characterize the genes harboring the variants, providing information on gene location, transcript structure, and protein-coding sequence. To elucidate potential disease associations of identified variants, public databases such as the Online Mendelian Inheritance in Man (OMIM) [20] and ClinVar [21] were extensively employed. OMIM catalogs human genes and genetic disorders, while ClinVar provides information about the clinical significance of genetic variants.

Furthermore, population frequency information was compiled from several large-scale population databases to distinguish pathogenic variants from common polymorphisms. These databases included the 1000 Genomes Project [22], the Exome Aggregation Consortium (ExAC) [23], the Genome Aggregation Database (gnomAD) (both exome and genome data), and the Exome Sequencing Project (ESP) [24]. These resources provide allele frequencies across diverse human populations.

For functional prediction of the potential impact of missense variants, in silico prediction tools were used. These included SIFT (Sorting Intolerant From Tolerant) [25], PolyPhen-2 (Polymorphism Phenotyping v2) [26], FATHMM (Functional Analysis Through Hidden Markov Models) [27], MutationTaster [28], MutationAssessor [29], and PROVEAN (Protein Variation Effect Analyzer) [30]. Missense variants were further annotated with Combined Annotation Dependent Depletion (CADD) scores [31], which provide a single integrated score that predicts the deleteriousness of a variant. The SIFT-indel tool [32] was applied to evaluate the predicted functional impact of InDels.

### 2.7. Variant Filtering and Classification

To explore the genetic basis of COVID-19 susceptibility and identify relevant variants, we started by examining genes specifically included in the “COVID-19 research (Version 1.142)” panel of the Genomics England PanelApp [33]. PanelApp is a useful online tool that organizes gene panels linked to specific clinical traits. From this targeted set, we then selectively investigated variants found in these prioritized genes to uncover their possible roles in the disease. A multi-step filtering process was implemented to enhance the clinical relevance of the identified variants. Initially, synonymous and intronic variants were excluded, as these are less likely to have a direct impact on protein function. Only exonic non-synonymous variants, which alter the amino acid sequence, and indel variants, which introduce insertions or deletions in the DNA sequence, were retained. Furthermore, variants exhibiting a minor allele frequency (MAF) of 1% or less in population databases, including gnomAD [34] and the 1000 Genomes Project [22], were retained. This step aimed to filter out common polymorphisms that are less likely to be disease-causing.

Variants were annotated using ClinVar [21] to leverage existing clinical knowledge and interpretations. The clinical significance of each filtered variant was then assessed and classified according to the American College of Medical Genetics and Genomics (ACMG) and the Association for Molecular Pathology (AMP) 2015 guideline [35]. This guideline provides a standardized framework for interpreting sequence variants.

Variant classification was performed using the InterVar software tool [36] and the Varsome platform [37]. InterVar is a tool that automates the application of the ACMG/AMP criteria. Varsome is a comprehensive database and tool that aids in variant interpretation. Based on the ACMG/AMP criteria, all variants were classified as benign (B), likely benign (LB), pathogenic (P), likely pathogenic (LP), or variants of uncertain significance (VUS).

### 2.8. Protein–Protein Interaction Network Analysis

A curated list of genes harboring pathogenic, likely pathogenic, and variants of uncertain significance (VUS) was subjected to protein–protein interaction (PPI) network analysis. This analysis was performed using the STRING database, version 12.0 [38]. STRING provides a comprehensive resource of known and predicted protein–protein interaction. The database integrates various sources of evidence, including experimental data, co-expression, and computational predictions, to assign confidence scores to interactions.

To obtain the interaction data, a downloadable tabular file (.tsv format) was acquired through the database’s “Exports” interface. The *Homo sapiens* (human) organism was specified, and interactions with a combined confidence score of 0.7 (high confidence) were selected to focus on the most reliable interactions. This dataset, containing a list of interacting protein pairs and their associated confidence scores, served as the foundation for visualizing the network.

Network visualization and further analysis were performed using Cytoscape version 3.10.1 [39], an open-source software platform specifically designed for the visualization and analysis of biomolecular interaction networks. The tabular data from STRING was imported into Cytoscape, where each protein was represented as a node, and the interactions between proteins were represented as edges. Hub genes within the network were identified using the cytoHubba plugin [40] in Cytoscape. Specifically, the degree algorithm, which ranks nodes based on their number of connections, was used to determine the most highly connected proteins (hub genes). The network was rearranged and visualized using the yFiles layout algorithms available in Cytoscape, which optimize the layout for clarity and readability.

### 2.9. Functional Enrichment Analysis

Functional enrichment analysis of the constructed protein–protein network was performed using the STRING database’s online analysis tool [38]. This analysis aimed to identify over-represented biological functions, pathways, and other annotations within the set of network genes.

The STRING database was used to enrich the network genes across a comprehensive range of functional categories, including: Biological Process (Gene Ontology), Molecular Function (Gene Ontology), Cellular Component (Gene Ontology), Reference Publications (PubMed), Local Network Cluster (STRING), KEGG Pathways, Reactome Pathways, WikiPathways, Disease-Gene Association (DISEASE), Tissue Expression (TISSUES), Subcellular Localization (COMPARTMENTS), Annotated Keywords (UniProt), Protein Domain and Feature (InterPro), and Protein Domain (SMART).

For each enriched term, the STRING analysis provides several statistical measures:

(1) **Count in-network:** The number of proteins in the user-provided network that are annotated with the term.
(2) **Strength:** The log10 of the observed-to-expected ratio, quantifies the magnitude of the enrichment effect.
(3) **Signal:** A weighted harmonic mean of the observed/expected ratio and the negative log10 of the False Discovery Rate (FDR), representing the overall enrichment significance.
(4) **False Discovery Rate (FDR):** A measure of the statistical significance of the enrichment, accounting for multiple testing.

For this study, Gene Ontology—Biological Process, Reactome Pathways, Disease-Gene Association, and Annotated Keywords (UniProt) categories were selected for enrichment representation, as these categories provide key insights into the biological functions, pathways, disease relevance, and protein features of the network genes. Enriched terms within each category were sorted based on their Signal value, prioritizing the most significant enrichments. To reduce redundancy, terms with a similarity score of 0.8 or greater (indicating substantial overlap in associated genes) were grouped together. The top 10 most significant terms from each selected category were then chosen for presentation, providing a concise overview of the key functional enrichments within the network.

## 3. Results

### 3.1. Clinical Characteristics of COVID-19 Patients

This exploratory study investigated the clinical characteristics of a cohort comprising 16 patients admitted to the hospital due to COVID-19 symptoms. The cohort included 9 female patients (56.3%) and 7 male patients (43.8%). The clinical outcomes varied, with 8 patients (50%) achieving full recovery and subsequent discharge, while 8 patients (50%) experienced fatal outcomes due to severe disease complications. The median duration of hospital stay for the entire cohort was 12 days, with an interquartile range (IQR) of 9 to 16 days. Among those requiring intensive care, the median length of stay in the intensive care unit (ICU) was 9 days (IQR: 5–12).

The median age of the patient cohort was 59 years (IQR: 50–69). The most frequently reported respiratory symptom was cough, present in 14 out of 16 patients (88%), followed by fever, which was observed in 10 patients (63%). Pre-existing comorbidities were common, with hypertension being the most prevalent, affecting 12 patients (75%). Diabetes mellitus was diagnosed in 5 patients (31%), and chronic kidney disease was present in 1 patient (6.3%). Notably, all patients (100%) received treatment involving immunosuppressive steroids and/or antiviral medications. The primary clinical characteristics of this patient cohort are summarized in Table 1.

### 3.2. Identification of COVID-19-Specific Variants in Cohort

**Figure 1:**
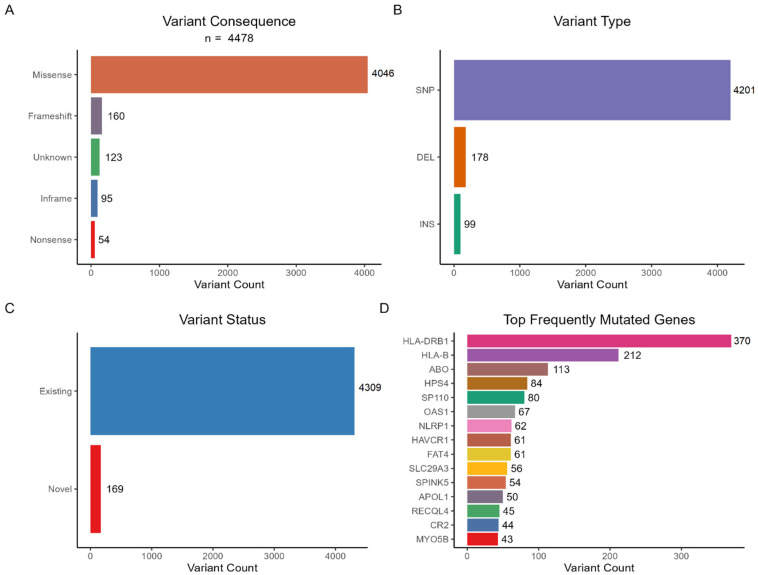
Characteristics of panel-based variants in the cohort. (**A**) Distribution of variant consequences. (**B**) Distribution of variant types. (**C**) Distribution of variant status. (**D**) Frequency of the top 15 mutated genes.

The “COVID-19 research (Version 1.142)” gene panel is an ongoing research effort aiming to catalog genes associated with susceptibility to viral infections, encompassing SARS-CoV-2 and other viruses. This panel categorizes genes based on the strength of evidence supporting their involvement. Genes with high levels of evidence and expert review are designated as “Green.” In the specified version of the panel, there were 697 entities, of which 461 genes were classified as “Green”. These 461 genes were used to prioritize variants identified in Whole-Exome Sequencing (WES) data. Following the filtering of synonymous and intronic variants, a total of 4478 variants were identified, distributed across 322 genes. The majority of these variants were missense substitutions (n = 4046, 90%). Additionally, the dataset included 160 frameshift variants, 95 inframe insertions/deletions (indels), and 54 nonsense variants. (Figure 1) The status of 123 variants could not be annotated. The variants were further categorized by type: 4201 were single-nucleotide polymorphisms (SNPs), 178 were deletions (DEL), and 99 were insertions (INS). Before filtering based on population frequency, 4309 identified variants were present in existing databases, while 169 variants were novel. The gene with the highest number of variants was *HLA-DRB1* (n = 370), followed by *HLA-B* (n = 212), *ABO* (n = 113), *HPS4* (n = 84), and *SP110* (n = 80). It is important to note that this variant count includes both common polymorphisms and rare variants, before frequency-based filtering.

### 3.3. Identification of Rare Candidate Variants in the Cohort

**Figure 2:**
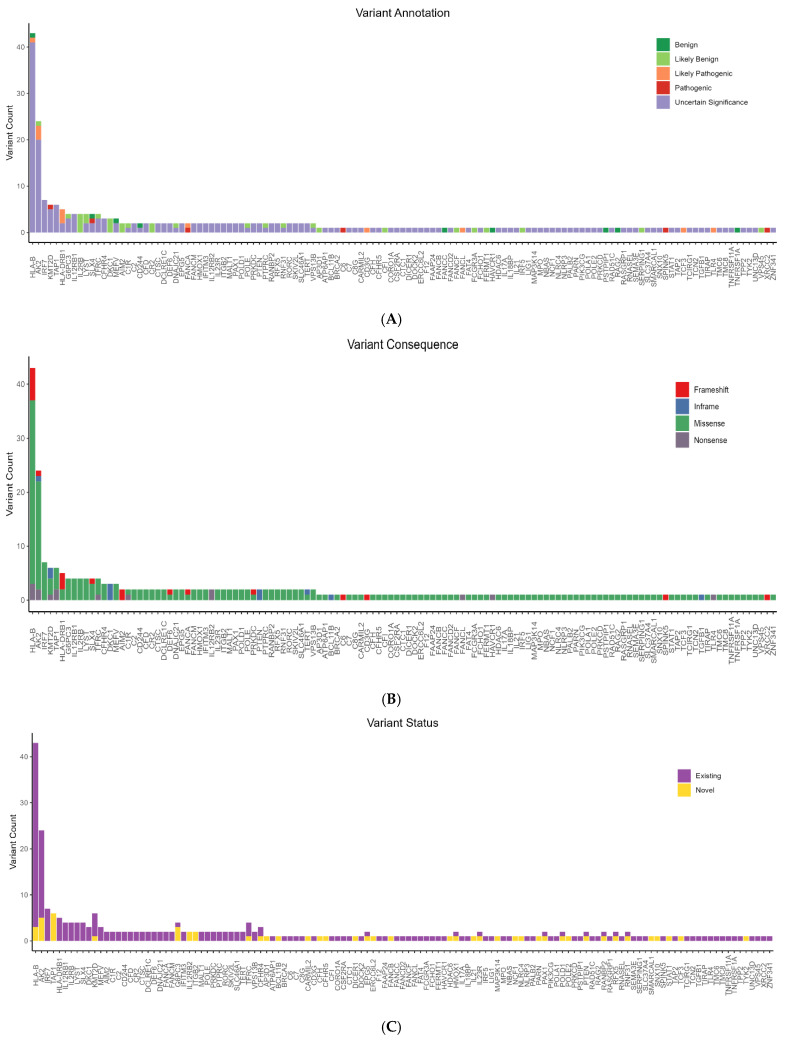
Categorization of rare variants identified in COVID-19 specific genes. (**A**) Variant ACMG classification. (**B**) Distribution of variant consequences. (**C**) Distribution of variant status.

Following population frequency-based filtering and annotation according to the American College of Medical Genetics and Genomics (ACMG) guidelines, 267 rare candidate variants were identified across 125 genes. Missense variants were the most frequent (n = 221, 83%), followed by frameshift variants (n = 20, 7.5%), nonsense variants (n = 15, 5.5%), and inframe insertions/deletions (n = 11, 4%). ACMG classification revealed that the majority of these variants were classified as variants of uncertain significance (VUS) (n = 210, 79%). Additionally, 30 variants were classified as likely benign (LB), 9 as benign (B), 12 as likely pathogenic (LP), and 9 as pathogenic (P). Of the total variants, 214 had been previously reported, while 53 were classified as novel. The *HLA-B* gene exhibited the highest number of rare candidate variants (n = 43), followed by *AK2* (n = 24), *IRF7* (n = 7), *KMT2D* (n = 6), *TAP1* (n = 6), and *HLA-DRB1* (n = 5). Several genes harbored multiple novel variants, including *TAP1* (n = 6), *AK2* (n = 5), *G6PC3* (n = 3), *HLA-B* (n = 3), *IL12RB2* (n = 2), and *ITGB2* (n = 2). The distribution of gene candidate variants across different ACMG categories is illustrated in Figure 2.

**Figure 3:**
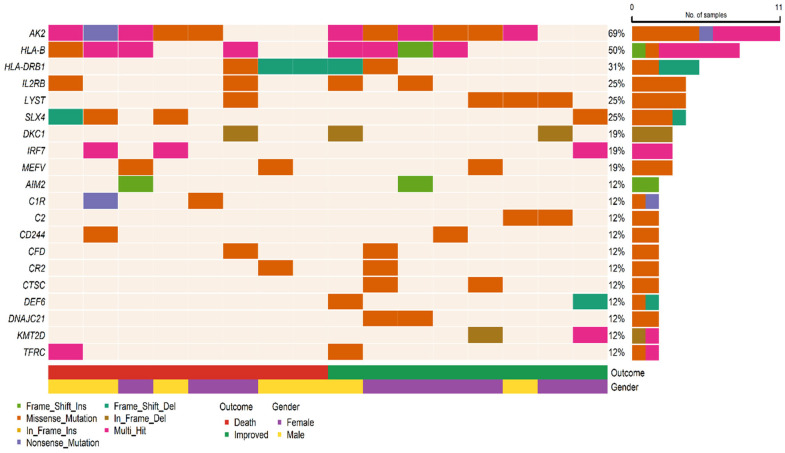
Comutation plot illustrating the co-occurrence of top variants across patient samples. Columns represent individual patient samples, and rows represent genes.

Analysis of patient samples showed that *AK2* gene variants were present in 11 samples (69%). *HLA-B* variants were observed in half of the patients. *HLA-DRB1*, *IL2RB*, *LYST*, and *SLX4* variants were each present in 31%, 25%, 25%, and 25% of the samples, respectively. The distribution of these variants among patients is depicted as a computational plot in Figure 3.

### 3.4. Rare Candidate Pathogenic Variants in the Cohort

A total of 18 rare candidate variants were classified as either pathogenic (n = 6) or likely pathogenic (n = 12) within the study cohort. The AK2 variant (p.185delinsTS) was recurrent, observed in two patients, and the HLA-DRB1 variant (p.E198Gfs18) was present in three patients. Additional variants classified as pathogenic included FANCA (p.E1254Gfs23), KMT2D (p.Q3735X), SLX4 (p.Q365Sfs32), SPINK5 (p.K824Efs4), and XRCC2 (p.L117Ffs6). Variants classified as likely pathogenic were found in AK2 (p.185delinsTS and p.I167Mfs8), CD3G (p.K71Nfs40), FANCA (p.K921I), FANCL (p.Q75X), HLA-B (p.T202Afs18), HLA-DRB1 (p.E198Gfs*18), TCF3 (p.V595I), and TLR4 (p.R731X). Notably, the TCF3 variant (p.V595I) represents a novel likely pathogenic variant not previously reported in existing databases. Among these, FANCA (p.E1254Gfs23), FANCL (p.Q75X), and HLA-B (p.T202Afs18) were observed to be homozygous, while all other identified variants were heterozygous (Table 2). To confirm the rarity of these findings and to ensure they are not common polymorphisms within the Saudi population, the frequencies of these variants were searched in the gnomAD Middle East polymorphism dataset. All of the identified variants were either absent from the database or had frequencies nearing 0%. Specifically, C6 (p.D627Tfs4) and CD3G (p.K71Nfs40) had frequencies of 0.03% and 0.02%, respectively, while TLR4 (p.R731X) and XRCC2 (p.L117Ffs6) showed frequencies of 0.02% and 0.01%, respectively, in the Middle Eastern population (Table 3). This population data reinforces the conclusion that the identified variants are genuinely rare and potentially disease-causing.

### 3.5. Protein–Protein Interaction Network Analysis

A protein–protein interaction (PPI) network was constructed to analyze the relationships among the identified genes. This network specifically included genes harboring pathogenic, likely pathogenic, and variants of uncertain significance (VUS). Genes with benign or likely benign variants were excluded from this analysis, as our focus was on variants with the potential to alter gene functionality.

We opted to include VUS in the network despite their lack of conclusive evidence for pathogenicity. By definition, VUS are “of uncertain significance” precisely because there is not enough data to definitively classify them as benign or pathogenic. This inherent uncertainty suggests they could still exert a functional impact on their respective proteins. Including these variants in the PPI network allowed us to explore whether the proteins they encode interact with other proteins already known to be involved in relevant disease pathways or biological processes. The resulting network comprised 108 genes.

The PPI network, generated using the STRING database, exhibited 108 nodes (representing the genes) and 394 edges (representing the interactions between the genes). The average node degree, a measure of the average number of interactions per gene, was 7.3. The average local clustering coefficient, which quantifies the tendency of nodes to cluster together, was 0.532. A PPI enrichment *p*-value of <1.0 × 10^−16^ was observed, indicating that the network possesses significantly more interactions than would be expected by chance, suggesting a biologically meaningful structure.

**Figure 4:**
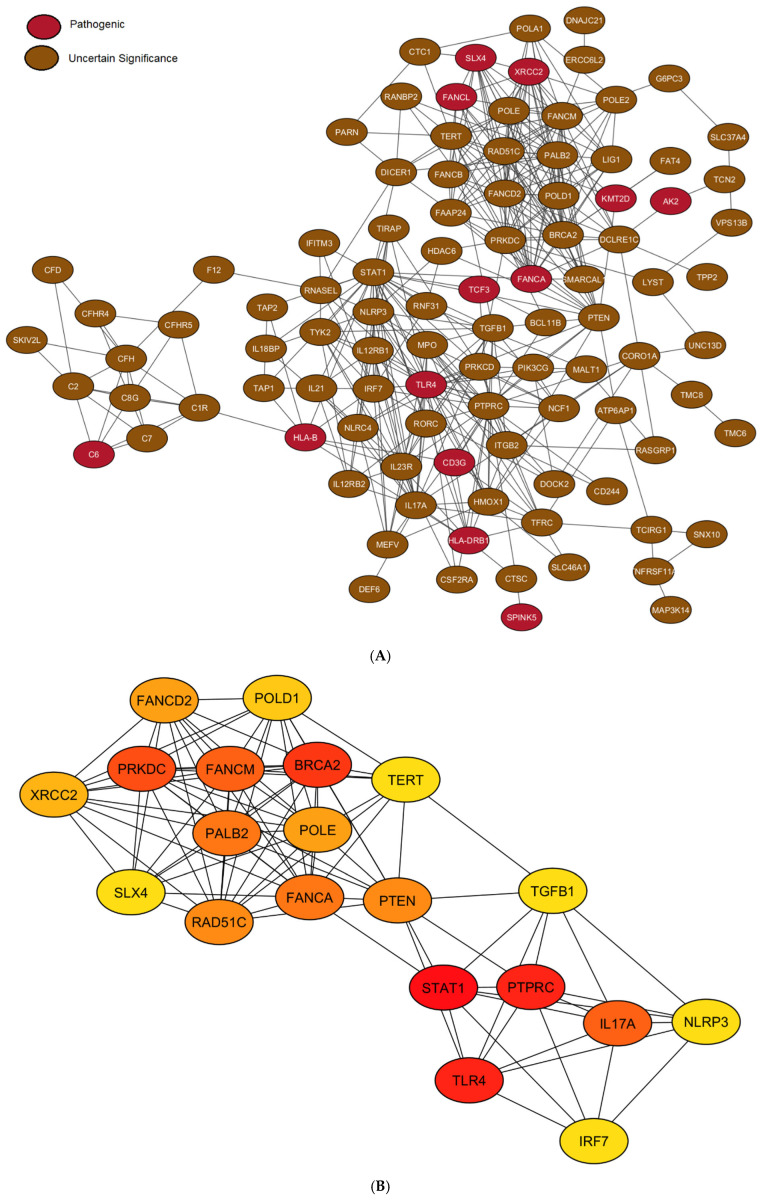
Network analysis of P/LP and VUS variants. (**A**) Complete network of P/LP and VUS genes. (**B**) Network of the top 20 hub genes, ranked by degree algorithm (red to yellow gradient).

Hub gene analysis, employing the degree algorithm to identify highly connected nodes, revealed key genes within the network. The top 20 hub genes, ranked in descending order of connectivity, were: *STAT1*, *TLR4*, *PTPRC*, *BRCA2*, *PRKDC*, *FANCM*, *IL17A*, *PALB2*, *FANCA*, *RAD51C*, *PTEN*, *FANCD2*, *POLE*, *XRCC2*, *POLD1*, *SLX4*, *TERT*, *NLRP3*, *IRF7*, and *TGFB1*. Notably, a substantial proportion of these hub genes are implicated in either DNA repair processes or inflammasome complexes, suggesting their potential importance in the context of the disease under investigation. The network and hub genes are illustrated in Figure 4.

### 3.6. Functional Enrichment Analysis

**Figure 5:**
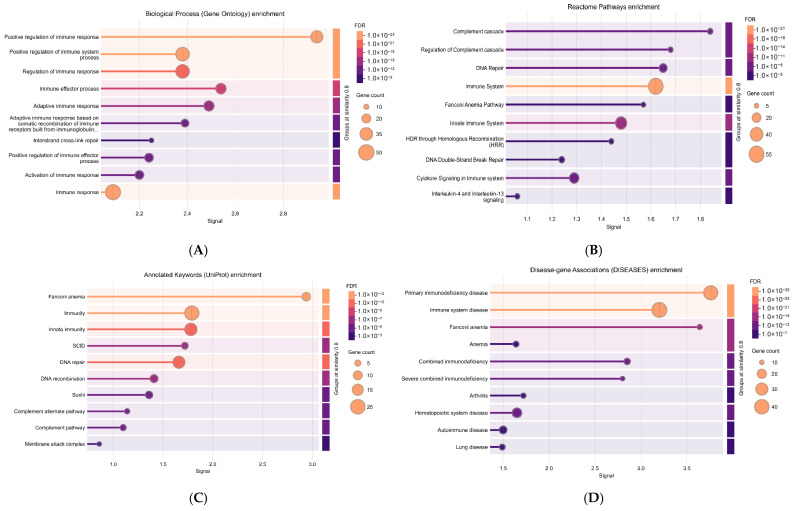
Lollipop plots of enriched terms, sorted by signal. Term groups with 80% similarity were merged. (**A**) Gene Ontology (GO) biological process enrichment. (**B**) Reactome pathway enrichment. (**C**) UniProt keyword enrichment. (**D**) Disease-gene association enrichment.

Functional enrichment analysis of the constructed protein–protein interaction network revealed that the identified genes are primarily enriched in pathways associated with immune deficiency and DNA repair. Gene Ontology (GO) Biological Process analysis showed significant enrichment in processes such as positive regulation of immune response (*p* = 2.05 × 10^−22^), immune effector process (*p* = 1.06 × 10^−16^), and adaptive immune response (*p* = 4.92 × 10^−16^). Reactome pathway analysis highlighted enrichment in DNA Repair (*p* = 1.79 × 10^−9^), Immune System (*p* = 3.93 × 10^−21^), Fanconi Anemia Pathway (*p* = 1.25 × 10^−6^), Innate Immune System (*p* = 1.11 × 10^−12^), and Homologous Recombination (HRR) (*p* = 1.92 × 10^−6^). Disease-gene association analysis, based on the DISEASES database (Jensen Lab), demonstrated enrichment in primary immunodeficiency disease (*p* = 8.24 × 10^−30^), Fanconi anemia (*p* = 2.06 × 10^−14^), immune system disease (*p* = 2.53 × 10^−29^), combined immunodeficiency (*p* = 8.20 × 10^−13^), and severe combined immunodeficiency (SCID) (*p* = 1.72 × 10^−11^) Figure 5.

Consistent with these findings, enrichment of UniProt keywords revealed associations with Fanconi anemia (*p* = 1.33 × 10^−11^), immunity (*p* = 2.06 × 10^−12^), innate immunity (*p* = 1.16 × 10^−10^), SCID (*p* = 5.39 × 10^−7^), and DNA repair (*p* = 8.08 × 10^−10^). Table 4 shows the functional enrichment of the candidate genes.

## 4. Discussion

Several factors contribute to the susceptibility towards SARS-CoV-2 infections and its severity including genetics, gender and comorbidity. Men generally experience more severe COVID-19 outcomes and higher mortality rates compared to women [41]. Such a disparity has been observed across multiple countries and populations, suggesting a biological basis for the difference. While infection rates may vary, the severity of the disease and the likelihood of death are consistently higher in men. Our study also reports mortality in male patients due to severe disease. Angiotensin-converting enzyme 2 serves as the primary entry receptor for SARS-CoV-2 into human cells. ACE2 has a protective effect on acute lung injury and acute respiratory distress syndrome [42]. It counteracts the effects of angiotensin II, a peptide that promotes inflammation and vasoconstriction. It converts angiotensin II into angiotensin 1–7, and thus promotes vasodilation, reduces inflammation, and protects against organ damage. SARS-CoV-2 binding to ACE2 leads to down-regulation of ACE2 expression, disrupting the balance of the RAS system, exacerbating lung injury and contribute to the pathogenesis of pulmonary hypertension and insufficiency. The disruption of the ACE2-mediated protective mechanisms may explain the progression to ARDS observed in severe COVID-19 cases. Estrogens are believed to up-regulate ACE2 expression, potentially contributing to the lower disease severity observed in pre-menopausal women [43]. This increased ACE2 expression could provide a larger reservoir of ACE2 to maintain the RAS-regulatory axis after viral infection. Such discrepancy based on gender can also be explained in the light of the theory that the X chromosome carries a greater concentration of immune-related genes [44]. Consequently, women typically exhibit stronger innate and adaptive immune responses compared to men, owing to the presence of two X chromosomes. In a retrospective case–control study, the association between COVID-19 severity (severe vs. asymptomatic/oligosymptomatic healed individuals) and HLA gene variants was analyzed by next-generation sequencing [45]. The study identified significant HLA alleles, SNPs and haplotypes in the *HLA-B*, *-C*, *-F*, *-DQA1*, *-DRB1*, and *-DRB5* genes associated with COVID-19 severity. Interestingly, these variants showed biological sex-related effects. Additionally, the haplotypes had a biological sex-specific impact on disease severity and markedly increased the risk of severe COVID-19.

The study cohort has shown substantial burden of comorbidity in COVID-19 patients including hypertension, ischemic heart disease and T2DM. Such comorbid conditions have been identified as significant determinants of COVID-19 as each of these independently increases the risk of adverse outcomes in COVID-19 patients [46]. The presence of these comorbidities significantly alters the clinical course of COVID-19, leading to a more complex and challenging disease management. The prevalence of hypertension among COVID-19 patients is substantial, with studies estimating that it affects a significant proportion of hospitalized individuals [47]. The presence of CAD compromises the heart’s ability to respond to the increased demands imposed by the viral infection, leading to a higher risk of complications and death. CAD reduces oxygen supply to the myocardium, making the heart more vulnerable to the effects of SARS-CoV-2 infection. CAD-related inflammation and endothelial dysfunction contribute to thromboembolic events in COVID-19, further worsening the prognosis [48]. T2DM impairs immune function, promotes chronic inflammation, and increases the risk of cardiovascular and renal complications, all of which contribute to the heightened vulnerability to COVID-19. Hyperglycemia promotes inflammation, microvascular obstruction, and the no-reflow phenomenon, further exacerbating poor outcomes [49]. Long-term renal outcomes in COVID-19 survivors with diabetes are concerning, with an increased risk of chronic kidney disease progression and end-stage renal disease [50].

Host genetic factors play a crucial role in determining the diverse responses observed following SARS-CoV-2 infection. Genetic variability is a significant determinant in both an individual’s susceptibility to SARS-CoV-2 infection and the severity of COVID-19 outcomes [9]. Using next-generation Whole-Exome Sequencing, a total of 4478 variants were identified, distributed across 322 genes within the cohort. The majority of these variants were missense substitutions along with frameshift variants, inframe insertions/deletions (indels), and nonsense variants. The variants were further categorized by types to include single-nucleotide polymorphisms (SNPs), deletions (DEL), and insertions (INS). The gene with the highest number of variants was *HLA-DRB1* followed by *HLA-B*, *ABO*, *HPS4* and *SP110* displaying both common polymorphisms and rare variants. Moreover, the *HLA-B* gene exhibited the highest number of rare candidate variants followed by *AK2*, *IRF7*, *KMT2D*, *TAP1*, and *HLA-DRB1*. Several genes harbored multiple novel variants, including *TAP1*, *AK2*, *G6PC3*, *HLA-B*, *IL12RB2*, and *ITGB2*.

Further, as mentioned our filtering process was designed to remove common variants using a 1% minor allele frequency (MAF) threshold in major public databases. This step is a key substitute for a population-matched control group in exploratory studies like ours. By removing variants with a MAF > 1%, we significantly increase the likelihood that our identified variants are indeed rare and not a common feature of any given population, including the Saudi population. To further substantiate this, we compared our identified variants with frequency data from a comprehensive public database, specifically focusing on the Middle Eastern population and the overall combined population. As shown in the attached Table 3, the frequencies of our identified variants are either zero or extremely low (below our 1% threshold).

For example:

- The AK2 variants, which were observed in multiple patients, have a frequency of 0 in the control population data.
- The HLA-DRB1 variant, a key finding, also has a frequency of 0.
- Other variants like those in C6, CD3G, and SPINK5 have very low frequencies (e.g., 0.0032, 0.0006, and 0.00022, respectively) and are completely absent in the Middle Eastern population data shown.

This data suggests that the variants we have identified are not common indigenous polymorphisms but are indeed rare. Therefore, their presence in our cohort of severe COVID-19 patients is a compelling finding that warrants further investigation.

Specific gene variants, including those within the HLA-DRB, HLA-B, and ABO blood group systems, have been linked to varying degrees of susceptibility and severity of COVID-19 [51,52]. Specific HLA-DRB alleles have been associated with either increased or decreased susceptibility to SARS-CoV-2 infection [53,54]. Human leukocyte antigen (HLA) genes, particularly HLA-DRB, play a pivotal role in the adaptive immune response, influencing recognition and response to viral infections. These genes encode proteins that present viral peptides to T cells, initiating an immune response. Certain HLA-DRB alleles may enhance the immune system’s ability to respond to SARS-CoV-2, thus reducing susceptibility to infection. Conversely, other alleles may impair this process, leading to increased susceptibility. HLA class II DRB1*01:01 and HLA class I B*35:01 may be protective against severe COVID-19 outcomes [55]. These alleles have been associated with reduced durations of COVID-19 symptoms and increased virus neutralizing capacity. Individuals carrying these alleles tend to have milder disease courses and are less likely to experience severe complications. Certain HLA-DRB alleles, such as HLA-DRB1*15, are linked to a more severe clinical course of COVID-19 [56]. This allele has been associated with increased duration of COVID-19 symptoms. Individuals carrying this allele may experience a more prolonged and challenging disease course, with a higher risk of developing severe complications. Certain HLA-B alleles, including B46:01, have also been associated with increased susceptibility to COVID-19 [51]. The HLA-B46:01 allele, for example, is thought to present fewer viral peptides, potentially leading to a weaker immune response and increased susceptibility. Studies on ABO blood typing in relation to the susceptibility to disease have shown individuals with blood group O may have a lower risk of SARS-CoV-2 infection, while those with blood group A may have a higher susceptibility [57]. The mechanisms underlying this potential protective effect may involve interactions between the ABO antigens and the virus or the host’s immune system. The non-O blood groups, which include A, B, and AB, have been linked to an elevated risk of thrombotic events, such as deep vein thrombosis (DVT) and pulmonary embolism (PE) [58]. These blood groups may influence levels of von Willebrand factor (VWF), a protein involved in blood clotting, potentially increasing the risk of thrombosis. The association between non-O blood groups and increased thrombosis may contribute to the severity of COVID-19, as thrombotic complications are a significant cause of morbidity and mortality in severe cases [59]. HPS4 (Hermansky–Pudlak Syndrome 4) is involved in the biogenesis of lysosome-related organelles. Hermansky–Pudlak Syndrome is a group of rare genetic disorders characterized by defects in lysosome-related organelles, which include melanosomes, platelet dense granules, and specialized immune cells [60]. HPS4, one of the genes associated with HPS, plays a critical role in the formation and function of these organelles. Mutations in HPS4 can affect immune cell function and inflammatory responses [61]. The SP110 protein, also known as Ipr1, is an interferon-inducible protein that plays a role in the innate immune response to viral infections. SP110 is expressed in various immune cells, including macrophages and dendritic cells, and is thought to enhance the transcription of interferon-stimulated genes (ISGs) [62]. SP110 enhances the transcription of interferon-stimulated genes (ISGs), playing a role in antiviral defense and thus the SP110 variants could influence susceptibility by modulating interferon responses.

Some important genes have been shown to have rare and novel variants in the cohort and are involved in a variety of cellular processes that are crucial for executing an effective immune response against viral infections. For instance, Interferon Regulatory Factor 7 (IRF7) is a key transcription factor that plays a central role in regulating the expression of type I interferons (IFNs), which are critical components of the innate immune response to viral infections [63]. Type I IFNs, including IFN-α and IFN-β, are cytokines that have potent antiviral activity, inhibiting viral replication and promoting the activation of immune cells. Upon viral infection, pattern recognition receptors (PRRs) such as Toll-like receptors (TLRs) and RIG-I-like receptors (RLRs) detect viral components and activate intracellular signaling cascades. These cascades lead to the phosphorylation and activation of IRF7, which then translocate to the nucleus and binds to IFN-stimulated response elements (ISREs) in the promoters of type I IFN genes. This binding triggers the transcription of IFN genes, leading to the production and secretion of type I IFNs. IRF7 plays a crucial role in both the induction and amplification of IFN signaling, ensuring a robust and sustained antiviral response [64]. The IRF7 c.887-2 A>C variant has been identified in critical/severe COVID-19 cases, suggesting that this specific variant may be associated with increased disease severity [65]. This variant, located in the IRF7 gene, may affect the splicing or stability of the IRF7 mRNA, leading to reduced IRF7 protein levels or impaired IRF7 function. Individuals carrying this variant may be more susceptible to developing severe COVID-19 due to a compromised IFN response. Transporter Associated with Antigen Processing 1 (TAP1) is a key component of the major histocompatibility complex (MHC) class I antigen-processing pathway, which is essential for presenting intracellular antigens to the immune system [66]. TAP1 transports peptides from the cytoplasm into the endoplasmic reticulum for loading onto MHC class I molecules, a crucial step in initiating the adaptive immune response against intracellular pathogens. Additionally, TAP1 is essential for presenting viral antigens to cytotoxic T lymphocytes (CTLs), which are critical for eliminating virus-infected cells. TAP1 variants might influence the effectiveness of vaccines that rely on the induction of CTL responses, highlighting the importance of considering host genetics in vaccine design and delivery. Adenylate Kinase 2 (AK2) is a crucial enzyme involved in maintaining cellular energy homeostasis by catalyzing the reversible transfer of phosphate groups between adenine nucleotides, specifically converting ADP and ATP [67]. AK2 also plays a role in mitochondrial function and apoptosis, processes that are intricately linked to cellular energy metabolism. AK2 deficiency can lead to immune dysfunction and increased susceptibility to infections, highlighting its importance in maintaining a healthy immune system. It has been demonstrated that AK2 is mutated in individuals with reticular dysgenesis, a severe form of immune deficiency in humans [68]. Lysine Methyltransferase 2D (KMT2D), also known as MLL4, is a histone methyltransferase that plays a crucial role in regulating gene expression through epigenetic modifications [69]. Specifically, KMT2D catalyzes the methylation of histone H3 at lysine 4 (H3K4me1), a modification that is associated with active enhancers and promoters. KMT2D plays a role in developmental processes, immune cell differentiation, and chromatin remodeling, highlighting its diverse functions in cellular biology. KMT2D has been shown to regulate inflammatory responses and immune homeostasis, through C-C motif chemokine ligand 2 (TAM chemotactic factor) mediated response [70]. A recent study has demonstrated KMT2D-altered integrin expression in B-cells and T-cells with distinct features of immunodeficiency [71]. Glucose-6-Phosphatase Catalytic Subunit 3 (G6PC3) is involved in glucose homeostasis and plays a critical role in neutrophil function, linking glucose metabolism to immune cell activity [72]. G6PC3 is an enzyme that catalyzes the final step in gluconeogenesis and glycogenolysis, the production of glucose from non-carbohydrate sources and the breakdown of glycogen, respectively. Mutations in G6PC3 can cause severe congenital neutropenia, a condition characterized by a deficiency of neutrophils, leading to increased susceptibility to bacterial and fungal infections [72]. G6PC3 is essential for proper neutrophil development, migration, and oxidative burst activity, all of which are critical for their ability to combat infections. Altered neutrophil function could contribute to increased inflammation and tissue damage in COVID-19, as neutrophils can release inflammatory mediators that contribute to the pathogenesis of severe disease [73]. While neutrophils are essential for clearing pathogens, their activation can also lead to the release of inflammatory mediators, such as cytokines and ROS, which can damage surrounding tissues. IL12RB2 is a subunit of the interleukin-12 (IL-12) receptor, which is crucial for Th1 immune responses and IFN-γ production, playing a pivotal role in cell-mediated immunity [74]. IL-12 is a heterodimeric cytokine composed of two subunits, IL-12p40 and IL-12p35, and its receptor is composed of two subunits, IL-12Rβ1 and IL-12Rβ2. The binding of IL-12 to its receptor activates intracellular signaling pathways that lead to the differentiation of T helper cells into Th1 cells, which are characterized by the production of IFN-γ. IL-12 is a key cytokine that promotes cell-mediated immunity and antiviral defense, stimulating the activity of NK cells and T lymphocytes [75]. ITGB2, also known as CD18, is a subunit of β2 integrins, which are essential for leukocyte adhesion and migration [76]. β2 integrins are a family of cell surface receptors that mediate the adhesion of leukocytes to endothelial cells and other cells in the body. ITGB2 plays a role in leukocyte recruitment to sites of inflammation and infection. Leukocytes express β2 integrins that bind to these adhesion molecules, allowing them to adhere to the endothelium and migrate into the surrounding tissues. It has been shown that elevated concentrations of ITGB2 were associated with long-term pulmonary complications in post-COVID-19 patients [77]. Functional enrichment analysis of the constructed protein–protein interaction network in our exploratory study revealed that the identified genes are primarily enriched in pathways associated with immune deficiency and DNA repair. These may also reflect on the significance of epistasis, where the effect of one gene is modified by another that may play a role in determining COVID-19 susceptibility and severity as different network of genes influence the disease status and progression.

## 5. Conclusions

Understanding the genetic variables that determine both susceptibility to COVID-19 and illness severity is critical. This pilot study, through its preliminary data derived from a limited sample size, offers important insights into the disease’s underlying mechanisms by identifying potential pathogenic gene variations linked to an increased incidence and severity of COVID-19. Continued study is needed to validate these findings, better understand the complex connections between genetic variables and COVID-19, and investigate potential targeted treatments based on individual genetic profiles. By understanding the significance of gene variations, a personalized approach can be established to reduce risk, improve preventive interventions, and improve outcomes for people who are more likely to contract severe COVID-19.

## 6. Limitations of the Study

The research presented in the manuscript corresponds to a pilot study. In this regard, it should be noted that the sample size is small. Moreover, the objective of the study was to identify novel and known genetic variations within the COVID-19 patient cohort in order to acknowledge host genetic factors in the risk and severity of the infection. Therefore, healthy controls were not included in the study. Moreover, the small sample size limits the statistical power and generalizability of our findings, making them mainly hypothesis-generating. Our use of Whole-Exome Sequencing (WES) is helpful for detecting exonic variants, but it does not enable thorough HLA typing or a complete analysis of the highly variable HLA region. This means we missed important non-exonic or full HLA allele variations. Finally, since this is an observational study, it cannot show direct causality, and we may not have fully addressed potential confounding factors. These limitations highlight the need for further studies using a large patient cohort and healthy controls to validate the plausible relationship of the genetic variants with the disease pathology.

## Figures and Tables

**Table 1:** Clinical characteristics of the patient cohort.

	COVID-19 Patients
Characteristic	n = 16
**Gender**	
Female	9 (56%) ^1^
Male	7 (44%)
**Age**	59 (50–69)
**Hypertension**	12 (75%)
**Diabetes**	
No	2 (13%)
Unknown	9 (56%)
Yes	5 (31%)
**Smoking**	
No	6 (38%)
Unknown	9 (56%)
Yes	1 (6.3%)
**Fever**	
No	3 (19%)
Unknown	3 (19%)
Yes	10 (63%)
**Cough**	
Unknown	2 (13%)
Yes	14 (88%)
**Chronic kidney disease**	
No	7 (44%)
Unknown	8 (50%)
Yes	1 (6.3%)
**Ischemic heart disease**	8 (50%)
**Immunosuppressive medications**	
No	2 (13%)
Unknown	3 (19%)
Yes	11 (69%)
**Hospital duration (Days)**	12 (9–16)
**ICU duration (Days)**	8 (5–12)
**Outcome**	
Death	8 (50%)
Improved	8 (50%)

^1^ n (%); Median (IQR).

**Table 2:** Pathogenic and likely pathogenic variants identified in the cohort.

Gene	Variation	Protein Change	Variant ID	Clinvar Classification	ACMG Classification ^1^	Interpreted Classification
AK2	NM_001319139.2:c.52_553insACATC5>C	p.*185delinsTS*	rs2035782928	-	LP	LP
AK2	NM_001319141.2:c.500_501insGACAT	p.I167Mfs*8	rs1398317449	LP	-	LP
C6	NM_000065.5:c.1879delG	p.D627Tfs*4	rs61469168	P	-	P
CD3G	NM_000073.3:c.205delA	p.K71Nfs*40	rs570768621	P/LP	LP	LP
FANCA	NM_000135.4:c.2762A>T	p.K921I	rs879255255	LP	-	LP
FANCA	NM_000135.4:c.3761_3762del	p.E1254Gfs*23	rs868273545	P	-	P
FANCL	NM_001114636.1:c.223C>T	p.Q75X	rs1693048371	-	LP	LP
HLA-B	NM_005514.8:c.604_605del	p.T202Afs*18	rs761596463	-	LP	LP
HLA-DRB1	NM_002124.3:c.593_612del	p.E198Gfs*18	rs140357311	-	LP	LP
KMT2D	NM_003482.4:c.11203C>T	p.Q3735X	rs1943059195	P	LP	P
SLX4	NM_032444.4:c.1093delC	p.Q365Sfs*32	rs1218169126	P	-	P
SPINK5	NM_001127698.2:c.2459dupA	p.K824Efs*4	rs587777750	P	-	P
TCF3	NM_001136139.4:c.1783G>A	p.V595I	Novel	-	LP	LP
TLR4	NM_138554.5:c.2191C>T	p.R731X	rs201670644	-	LP	LP
XRCC2	NM_005431.2:c.350dupT	p.L117Ffs*6	rs764640893	LP	P	P

^1^ ACMG classification is performed using the InterVar or Varsome tool. P—pathogenic; LP—likely pathogenic.

**Table 3:** Frequencies of the identified variants in the population-matched control group from the public databases.

Gene	Variation	Protein Change	Variant ID	Control Frequency (1000 Genome Combined Populations)	Control Frequency (GenomeAD Exome Combined Populations)	Control Frequency (GenomeAD Exome Middle East Populations)	Control Frequency (GenomeAD Genome Middle East Populations)
AK2	NM_001319139.2:c.52_553insACATC5>C	p.*185delinsTS*	rs2035782928	-	-	-	-
AK2	NM_001319141.2:c.500_501insGACAT	p.I167Mfs*8	rs1398317449	-	3.57 × 10^−5^	0	0
C6	NM_000065.5:c.1879delG	p.D627Tfs*4	rs61469168	0.0032	0.000314	0.000349	0
CD3G	NM_000073.3:c.205delA	p.K71Nfs*40	rs570768621	0.0006	5.87 × 10^−5^	0.000175	0
FANCA	NM_000135.4:c.2762A>T	p.K921I	rs879255255	-	-	-	-
FANCA	NM_000135.4:c.3761_3762del	p.E1254Gfs*23	rs868273545	-	6.84 × 10^−7^	0	0
FANCL	NM_001114636.1:c.223C>T	p.Q75X	rs1693048371	-	1.37 × 10^−6^	0	-
HLA-B	NM_005514.8:c.604_605del	p.T202Afs*18	rs761596463	-	-	-	0
HLA-DRB1	NM_002124.3:c.593_612del	p.E198Gfs*18	rs140357311	-	-	-	-
KMT2D	NM_003482.4:c.11203C>T	p.Q3735X	rs1943059195	-	-	-	-
SLX4	NM_032444.4:c.1093delC	p.Q365Sfs*32	rs1218169126	-	5.47 × 10^−6^	0	0
SPINK5	NM_001127698.2:c.2459dupA	p.K824Efs*4	rs587777750	-	0.00022	0	0
TCF3	NM_001136139.4:c.1783G>A	p.V595I	Novel	-	-	-	-
TLR4	NM_138554.5:c.2191C>T	p.R731X	rs201670644	-	5.82 × 10^−5^	0.001057	0
XRCC2	NM_005431.2:c.350dupT	p.L117Ffs*6	rs764640893	-	-	-	-

“-“ indicates the absence of a variant in the respective database.

**Table 4:** Functional enrichment analysis of selected genes, categorized by Gene Ontology (GO) biological process, Reactome pathways, UniProt keywords, and disease-gene associations.

Term Category	Enriched Term	Strength	Signal	p_adj_	Enriched Genes
**Diseases**	Primary immunodeficiency disease	1.17	3.76	8.2 × 10^−30^	UNC13D, CORO1A, MEFV, MPO, CFHR5, IL12RB2, NCF1, RFX5, C2, RASGRP1, PRKDC, IL23R, TMC8, PRKCD, CARMIL2, NLRP3, TAP1, BCL11B, HLA-DRB1, STAT1, CFH, ATP6AP1, TAP2, ZNF341, DCLRE1C, IRF7, ITGB2, HLA-B, PTPRC, DOCK2, CD3G, TYK2, IL18BP, TMC6, IL12RB1, IL21, IL17A, AK2
	Fanconi anemia	1.90	3.64	2.1 × 10^−14^	PALB2, FANCM, FANCD2, SLX4, RAD51C, XRCC2, BRCA2, FANCA, FANCL, TNFRSF11A, FANCB
	Immune system disease	1.05	3.20	2.5 × 10^−29^	UNC13D, CORO1A, MEFV, MPO, CFHR5, IL12RB2, NCF1, RFX5, C2, RASGRP1, PRKDC, RNF31, IL23R, TMC8, PRKCD, CARMIL2, NLRP3, TAP1, BCL11B, HLA-DRB1, STAT1, CFH, ATP6AP1, TAP2, ZNF341, DCLRE1C, LYST, FAT4, IRF7, ITGB2, HLA-B, PTPRC, DOCK2, CD3G, TYK2, IL18BP, TMC6, IL12RB1, IL21, IL17A, MALT1, AK2
	Combined immunodeficiency	1.49	2.85	8.2 × 10^−13^	CORO1A, RFX5, PRKDC, CARMIL2, TAP1, BCL11B, TAP2, DCLRE1C, ITGB2, PTPRC, DOCK2, CD3G, AK2
	Severe combined immunodeficiency	1.72	2.80	1.7 × 10^−11^	CORO1A, RFX5, PRKDC, TAP1, BCL11B, TAP2, DCLRE1C, PTPRC, CD3G, AK2
**GO** **Process**	Positive regulation of immune response	1.08	2.94	2.0 × 10^−22^	TGFB1, CFHR5, C6, C2, RASGRP1, PRKDC, RNF31, IL23R, C7, PRKCD, NLRP3, HLA-DRB1, CFHR4, CFH, C8G, TLR4, TAP2, TFRC, TIRAP, IRF7, ITGB2, NLRC4, HLA-B, PTPRC, CD3G, TYK2, C1R, CFD, IL12RB1, FCHO1, IL21, IL17A, MALT1
	Immune effector process	1.08	2.54	1.1 × 10^−16^	UNC13D, CORO1A, MPO, CTSC, CFHR5, C6, TCIRG1, C2, RASGRP1, C7, RORC, PRKCD, CFHR4, CFH, CD244, C8G, TLR4, TAP2, LYST, IRF7, PIK3CG, DOCK2, C1R, CFD, IL21
	Adaptive immune response	1.09	2.49	4.9 × 10^−16^	UNC13D, TGFB1, CTSC, C6, TCIRG1, C2, C7, RORC, PRKCD, TAP1, HLA-DRB1, CD244, C8G, TLR4, TAP2, DCLRE1C, IRF7, HLA-B, PIK3CG, CD3G, IL18BP, C1R, TNFRSF11A, IL17A
	Adaptive immune response based on somatic recombination of immune receptors built from immunoglobulin superfamily domains	1.24	2.39	2.4 × 10^−12^	UNC13D, TGFB1, CTSC, C6, TCIRG1, C2, C7, RORC, PRKCD, HLA-DRB1, C8G, TLR4, TAP2, IRF7, IL18BP, C1R
	Positive regulation of immune system process	0.92	2.38	2.0 × 10^−22^	UNC13D, HMOX1, CORO1A, TGFB1, CTSC, CFHR5, IL12RB2, TCF3, C6, C2, RASGRP1, PRKDC, RNF31, IL23R, C7, PRKCD, NLRP3, HLA-DRB1, CFHR4, CFH, CD244, C8G, TLR4, TAP2, TFRC, TIRAP, IRF7, ITGB2, NLRC4, HLA-B, PTPRC, CD3G, TYK2, C1R, CFD, IL12RB1, FCHO1, IL21, IL17A, MALT1
**Reactome**	Complement cascade	1.44	1.84	5.2 × 10^−8^	CFHR5, C6, C2, C7, CFHR4, CFH, C8G, C1R, CFD
	Regulation of complement cascade	1.47	1.68	3.0 × 10^−7^	CFHR5, C6, C2, C7, CFHR4, CFH, C8G, C1R
	DNA repair	1.00	1.65	1.8 × 10^−9^	POLE2, PALB2, LIG1, FANCM, FANCD2, SLX4, PRKDC, POLE, RAD51C, XRCC2, DCLRE1C, BRCA2, FANCA, FANCL, FAAP24, POLD1, FANCB
	Immune system	0.69	1.62	3.9 × 10^−21^	UNC13D, HMOX1, MEFV, TGFB1, MPO, CTSC, CFHR5, IL12RB2, C6, TCIRG1, RANBP2, NCF1, C2, RASGRP1, PRKDC, IL23R, C7, RORC, PRKCD, NLRP3, TAP1, HLA-DRB1, STAT1, CFHR4, CFH, RNASEL, C8G, PTEN, TLR4, TAP2, TPP2, TIRAP, IRF7, ITGB2, IFITM3, NLRC4, CSF2RA, HLA-B, PTPRC, PIK3CG, DOCK2, CD3G, TYK2, IL18BP, C1R, TMC6, TNFRSF11A, CFD, IL12RB1, MAP3K14, IL21, IL17A, MALT1
	Fanconi anemia pathway	1.54	1.57	1.2 × 10^−6^	FANCM, FANCD2, SLX4, FANCA, FANCL, FAAP24, FANCB
**UniProt Keywords**	Fanconi anemia	1.89	2.94	1.3 × 10^−11^	PALB2, FANCD2, SLX4, RAD51C, XRCC2, BRCA2, FANCA, FANCL, FANCB
	Immunity	0.91	1.79	2.1 × 10^−12^	MEFV, C6, C2, PRKDC, IL23R, C7, NLRP3, TAP1, HLA-DRB1, CFH, CD244, C8G, TLR4, TAP2, DCLRE1C, TIRAP, IRF7, IFITM3, NLRC4, HLA-B, PIK3CG, CD3G, C1R, CFD
	Innate immunity	1.00	1.78	1.2 × 10^−10^	MEFV, C6, C2, PRKDC, IL23R, C7, NLRP3, CFH, CD244, C8G, TLR4, TIRAP, IRF7, IFITM3, NLRC4, HLA-B, C1R, CFD
	SCID	1.74	1.72	5.4 × 10^−7^	RFX5, PRKDC, BCL11B, DCLRE1C, PTPRC, AK2
	DNA repair	0.98	1.66	8.1 × 10^−10^	PALB2, LIG1, FANCM, FANCD2, SLX4, PRKDC, POLE, RAD51C, XRCC2, DCLRE1C, BRCA2, FANCA, FANCL, FAAP24, POLD1, FANCB, ERCC6L2

## Data Availability

All data supporting the reported results can be found in the Prince Fahad Bin Sultan Chair for Biomedical Research, Faculty of Applied Medical Sciences, University of Tabuk, Tabuk, Saudi Arabia.
